# Exercise and load modification versus corticosteroid injection versus ‘wait and see’ for persistent gluteus medius/minimus tendinopathy (the LEAP trial): a protocol for a randomised clinical trial

**DOI:** 10.1186/s12891-016-1043-6

**Published:** 2016-04-30

**Authors:** Rebecca Mellor, Alison Grimaldi, Henry Wajswelner, Paul Hodges, J. Haxby Abbott, Kim Bennell, Bill Vicenzino

**Affiliations:** School of Health and Rehabilitation Sciences, The University of Queensland, Brisbane, Qld 4072 Australia; Physiotec, 23 Weller Road, Tarragindi, Qld 412 Australia; Department of Physiotherapy & Lifecare Physiotherapy, LaTrobe University, Bundoora, VIC 3086 Australia; NHMRC Centre of Clinical Research Excellence in Spinal Pain, Injury and Health, School of Health and Rehabilitation Sciences, The University of Queensland, Brisbane, Qld 4072 Australia; Centre for Musculoskeletal Outcomes Research, Dunedin School of Medicine, University of Otago, Dunedin, NZ 9054 New Zealand; Centre for Health, Exercise and Sports Medicine, Department of Physiotherapy, University of Melbourne, Carlton, VIC 3053 Australia

**Keywords:** Gluteal tendinopathy, Greater trochanteric pain syndrome, Corticosteroid injection, Physiotherapy

## Abstract

**Background:**

Lateral hip pain is common, particularly in females aged 40–60 years. The pain can affect sleep and daily activities, and is frequently recalcitrant. The condition is often diagnosed as trochanteric bursitis, however radiological and surgical studies have revealed that the most common pathology is gluteus medius/minimus tendinopathy. Patients are usually offered three treatment options: (a) corticosteroid injection (CSI), (b) physiotherapy, or (c) reassurance and observation. Research on Achilles and patellar tendons has shown that load modification and exercise appears to be more effective than other treatments for managing tendinopathy, however, it is unclear whether a CSI, or a load modification and exercise-based physiotherapy approach is more effective in gluteal tendinopathy. This randomised controlled trial aims to compare the efficacy on pain and function of a load modification and exercise-based programme with a CSI and a ‘wait and see’ approach for gluteal tendinopathy.

**Methods:**

Two hundred one people with gluteal tendinopathy will be randomly allocated into one of three groups: (i) CSI; (ii) physiotherapist-administered load modification and exercise intervention; and (iii) wait and see approach. The CSI therapy will consist of one ultrasound (US) guided CSI around the affected tendons and advice on tendon care. Education about load modification will be delivered in physiotherapy clinics and the exercise programme will be both home-based and supervised. The group allocated the wait and see approach will receive basic tendon care advice and reassurance in a single session by a trial physiotherapist. Outcomes will be evaluated at baseline, 4, 8, 12, 26 and 52 weeks using validated global rating of change, pain and physical function scales, psychological measures, quality of life and physical activity levels. Hip abductor muscle strength will be measured at baseline and 8 weeks. Economic evaluation will be performed to investigate the cost-effectiveness of the active interventions compared with the wait and see approach. Analyses will be conducted on an intention-to-treat basis using logistic and linear mixed regression models and the economic evaluation will report incremental cost-utility ratios. The trial reporting will comply with CONSORT guidelines.

**Discussion:**

This study will provide clinicians with directly applicable evidence of the relative efficacy of three common approaches to the management of gluteal tendinopathy.

**Trial registration:**

Australia New Zealand Clinical Trials Registry ACTRN12612001126808. Date Registered: 22/10/2012.

**Electronic supplementary material:**

The online version of this article (doi:10.1186/s12891-016-1043-6) contains supplementary material, which is available to authorized users.

## Background

Gluteal tendinopathy or greater trochanteric pain syndrome is a debilitating condition, characterised by pain situated at or around the greater trochanter of the hip, and tenderness on palpation. Although traditionally considered to be trochanteric bursitis, more advanced imaging and surgical procedures in people with lateral hip pain have revealed a primary pathology of insertional gluteus medius or minimus tendinopathy, with bursal distention generally a concomitant finding [1–3]. Magnetic Resonance Imaging (MRI) is very effective in recognizing partial and full thickness tears of the tendons of gluteus medius and minimus, tendon calcification and fatty muscle atrophy [2]. For the purposes of this paper we will refer to the condition of gluteus medius tendinopathy, and/or gluteus minimus tendinopathy, with or without bursal distention, as gluteal tendinopathy.

The condition is more frequent in women aged 40–60 years [4], and various studies have described its prevalence as ranging from 10 to 25 % of the general population [5, 6]. A recent study of general medical practice in the Netherlands found gluteal tendinopathy to have the highest prevalence (4.22 per 1000 person years) and incidence (3.29 per 1000 person years) of all presenting lower limb tendinopathies [7]. The impact of gluteal tendinopathy can be substantial. The pain experienced in side lying often creates significant sleep disturbance, and the pain commonly experienced with walking and stair climbing usually results in reduction of physical activity levels, which could be expected to have negative implications for general health and well-being, as well as quality of life and employment status [8].

It is proposed that abnormal hip biomechanics may predispose to gluteal tendinopathy [9], and although commonly diagnosed in sedentary, overweight people [5], it is also often seen in runners, possibly due to both poor training habits and technique [10]. It has been hypothesised that gluteal tendinopathy, with or without bursitis, occurs due to compressive impingement of these structures onto the underlying greater trochanter by the iliotibial band (ITB) as the hip moves into adduction [11, 12]. In an upright weight bearing posture, such as walking, weakness of the hip abductor muscles will result in lateral pelvic tilt or ipsilateral shift in single leg stance, and hip adduction, which will contribute to compression of the tendons between the greater trochanter and the thick fascia of the ITB.

The literature reflects an assumption that tight lateral structures are a key issue for the development of gluteal tendinopathy. Despite a lack of evidence of such tightness, the conservative management approach commonly suggested is stretching [13]. Compression as a primary aetiological factor is consistent with current theories for development of insertional tendinopathy [14, 15], and failure to control compression in a treatment protocol for insertional Achilles tendinopathy has been reported to result in poor outcomes [16]. The piriformis and iliotibial band stretches frequently prescribed [13] involve sustaining the hip in forced adduction, which is a position of high compressive loads on the gluteal tendons [17]. We hypothesise that a specific focus on hip abductor muscle function and avoidance of compressive loads on the tendons will provide a better approach to achieve effective treatment outcomes.

A range of conservative management approaches are generally recommended, including anti-inflammatory medications, rest, ice, heat, stretching, strengthening, ultrasound, shock-wave therapy (SWT), and local corticosteroid injection (CSI). Surgery is usually reserved for when the condition has become refractory and conservative measures have failed. However, optimal management of gluteal tendinopathy remains unclear. Reports concerning management of this condition predominantly relate to CSI and surgery. Early response to CSI in patients with lateral hip pain is reported to be very good, with 70–75 % of patients reporting a significant improvement at 1 month post injection in a randomised clinical trial (RCT) [13, 18]. However, at 3–4 months post injection, researchers have reported only 41–55 % positive response [13, 19]. At 12 months an RCT showed no difference between a CSI and a group that adopted a wait and see policy [19]. In the only clinical trial (non-randomised) to date to compare CSI to home exercise, the success rates at 1 month were 75 and 7 % respectively, but by 15 months were 48 % for CSI and 80 % for the home exercise group [13], demonstrating only short term benefits of CSI. These studies of CSI highlight the typical trajectory of delayed healing after a short term improvement, which in some studies is also coupled with substantial recurrence rates [20].

Most patients and their medical practitioners understandably seek a positive early response. Although Rompe et al report patient satisfaction with a slow rate of improvement in the early stages, a 7 % success rate for home exercise after 1 month [13] is not convincing evidence to recommend exercise to a patient who would otherwise have a 75 % chance of a successful early outcome with CSI. In contrast, outcomes from exercise and manual therapy for a comparable tendinopathy at the elbow (tennis elbow or lateral elbow tendinopathy, with similar age demographic) are comparable to those from CSI at 6 weeks (65 and 78 % success rates respectively) and superior to adopting a wait and see approach [21]. We speculate that the low success rates at 1 month reported by Rompe et al., in contrast to the high early success rates reported by Bissett et al., is the non-specific nature of the prescribed exercises (i.e. not targeting the muscles specifically affected by tendinopathy, using stretches that increase compression), a lack of specific load modification and management strategies related to tendon healing, and a low level of supervision of the home-based exercise programme.

The contemporary non-operative approach to effectively managing tendinopathy involves specific exercise for the affected muscles and load management in the form of advice and practical strategies [15]. This approach has yet to be rigorously tested in an RCT. We propose to test different conservative management options for gluteal tendinopathy by comparing an exercise and load management approach supervised by physiotherapists, CSI and the adoption of a wait and see approach.

Our primary hypotheses are that:

- HI: Both the exercise and load management programme and the CSI will be superior to a wait and see approach in terms of treatment success rates based on global rating of change and reductions in pain at 8 weeks.
- H2: The exercise and load management programme will be superior to CSI in terms of treatment success rates and reductions in pain at 52 weeks.

The secondary objectives are to: compare an exercise and load management programme with CSI on outcomes including pain, function, hip abductor muscle strength, psychological measures, quality of life and physical activity levels; evaluate the effects of the two treatments compared to a wait and see approach on these outcomes; determine the cost-effectiveness of the two treatments at 52 weeks; compare the adverse events associated with both treatments.

## Methods

This is a pragmatic, assessor-blinded, RCT conducted in medical and physiotherapy clinics in Brisbane and Melbourne, Australia. In people with persistent gluteal tendinopathy, it compares the effect of CSI, a physiotherapy supervised load management, education and exercise programme and a wait and see approach for gluteal tendinopathy over 12 months. The trial protocol will permit its reporting in line with the CONSORT guidelines [22].

### Participants

Lateral hip pain, which is a symptom of gluteal tendinopathy, might also present with other pathologies, such as trochanteric bursitis, osteoarthritis, referred lumbar spine pain, and femoral stress fracture [23]. As the load modification and exercise programme in this trial has been designed specifically to address gluteal tendinopathy, it is important that other differential diagnoses for lateral hip pain are excluded.

#### Recruitment details

We will recruit people from the community in both Brisbane and Melbourne via advertisements in University News, social media and local newspapers. Initial contact will be by phone or electronic media at which time a preliminary screening for suitability will occur. If the phone interview indicates potential eligibility, the volunteer will attend the trial centre for a physical examination, which will assess against specific selection criteria. Those who meet these criteria will then undergo diagnostic imaging to confirm eligibility prior to being included in the trial.

#### Selection criteria

We will include participants between the ages of 35 and 70 years who have experienced lateral hip pain for at least 3 months, of an intensity of ≥4/10 on an 11-point numeric rating scale on most days of the last 3 months. Table 1 outlines the selection criteria for inclusion into the study. These criteria were based on a previous study [13].

**Table 1:** Inclusion and Exclusion Criteria

Inclusion criteria
Lateral hip pain, worst over the greater trochanter, present for a minimum of 3 months
Age 35–70 years
Pain at an average intensity of ≥4 out of 10 on most days of the week.
Tenderness on palpation of the greater trochanter
Reproduction of pain on at least one of five diagnostic clinical tests (FABER test, Static muscle contraction in FABER position, FADER test, Adduction test, Static muscle contraction in Adduction position i.e. resisted abduction) or single leg stand
Demonstrated tendon pathology on MRI (see Table 2 for criteria)
Exclusion Criteria
Previous cortisone injection in the region of the lateral hip in the last 12 months
Physiotherapy intervention or regular appropriate Pilates in the last 3 months
Lumbar spine or lower limb surgery in the previous 6 months
Any known advanced hip joint pathology where groin pain is the primary complaint and/or where groin pain is experienced at an average intensity of ≥2 on most days of the week, or Kellgren-Lawrence score of >2 (mild) on XRay.
Where range of pure hip joint flexion is <90°
Significant signs of lumbar pathology
Known advanced knee pathology or restricted range of knee motion (must have minimum 90° flexion and full extension)
Any systemic diseases affecting the muscular or nervous system, and uncontrolled diabetes
Malignant tumour
Systemic inflammatory disease
Any factors that would preclude the participant from having an MRI (e.g. pacemaker, metal implants, pregnancy, claustrophobia)
If the participant is involved in a legal/workcover/TAC or other injury claim
If the participant is unable to commit to an 8 week exercise programme with twice weekly supervised sessions
Fear of needles (trypanophobia)
If the participant is unable to write, read or comprehend English

As clinical tests to diagnose gluteal tendinopathy appear to have limited validity [24], we have included a small battery of clinical tests that have been considered to be most provocative in reproducing symptoms of gluteal tendinopathy [23]. To be eligible, the participant must experience pain on direct palpation of the gluteal tendons’ insertion on the greater trochanter. They must also test positive (reproduction of trochanteric pain) to at least one of the following clinical tests: the Hip FADER (passive) test, static muscle test in the FADER position, the FABER (Patrick’s) test, passive hip adduction in side lying (ADD), a static muscle contraction in the ADD test position, and a Single Leg Stance on the affected leg for 30 s.

A. *Hip FADER* – With the patient supine, the hip is passively flexed to 90°, adducted and externally rotated to end of range (FADER = Flexion/Adduction/External Rotation). The pain Numeric Rating Scale (NRS) and area of pain is recorded. This test positions the ITB over the greater trochanter and places the Gluteus Medius (GMed) and Gluteus Minimus (GMin) tendons under tension while being compressed against the greater trochanter by the overlying fascia of the ITB. The test is only recorded as positive if the pain (≥2/10) is experienced over the lateral hip.
B. *Hip FADER with Static muscle test* (internal rotation) at end of range *(FADER-R)*. In the FADER position, the participant actively resists an external rotation force – i.e. performs static internal rotation (IR). At 90° hip flexion all portions of GMed and GMin are internal rotators [25]. This test requires the participant to activate these muscles, and therefore place further tension across their tendons, while they are in a compressed state. Again, a positive result refers to reproduction of pain at the lateral hip. As clinical features of gluteal tendinopathy include pain reproduction with elongation and compression of the involved tendons, as well as active contraction of these tendons, these two tests together may have improved diagnostic accuracy. This test is a modification of the resisted external de-rotation test, which has been reported to have 88 % sensitivity and 97.3 % specificity [26].
C. *Hip FABER* – (FABER = Flexion/Abduction/External Rotation). The lateral malleolus of the test leg is placed above the patella of the opposite side, the pelvis is stabilised via the opposite anterior superior iliac spine (ASIS) and the knee is passively lowered into abduction and external rotation. This test places the anterior portions of the GMed and GMin on tensile load. A positive pain response is usually felt in the lateral hip region. Lateral hip pain with a FABER test has been shown to have a high sensitivity, specificity, positive and negative predictive value (82.9, 90, 94.4 and 72 % respectively) for differentiating the diagnosis for greater trochanteric pain syndrome from hip osteoarthritis [27].
D. *Passive Hip Adduction in Side Lying (ADD)* – The participant is placed in side-lying, with the underneath hip and knee flexed 80–90°, and the uppermost leg supported by the examiner with the knee extended, in neutral rotation, and the femur in line with the trunk. The anterior superior iliac spines are aligned vertically in the frontal plane. The examiner passively moves the hip through a pure frontal plane motion into end range hip adduction with overpressure, while stablilising the pelvis with the other hand. This test places the lateral insertions of the gluteal tendons under compressive load, and a positive response is felt over the lateral hip. This is based on Ober’s test, which has been reported as having a high specificity (95 %), but a low sensitivity (41 %) and low negative predictive value (45.2 %) [27].
E. *ADD with resisted isometric abduction (ADD-R)* – In the ADD test end position, the participant is asked to push the thigh up, against the resistance of the examiner’s hand at the lateral knee. This test places tensile load on the compressed tendons, with pain elicited over the lateral hip.
F. *Single Leg Stance for 30 s (SLS)* – the participant stands side-on to a wall with one finger touching the wall at shoulder height for balance, then lifts the foot closest to the wall, maintaining single leg stance for up to 30 s. The participant is asked to immediately report the development of pain by pointing to the area of pain. If the region of the greater trochanter is indicated, the timer is stopped, the test ceased and recorded as positive. This time is reported, as well as the intensity of the pain. The single leg stance test has been shown to have good sensitivity and specificity (100 and 97.3 % respectively) [26] for the diagnosis of tendinopathy and bursitis in people with MRI-documented gluteal tendinopathy.

In addition to these tests, the physical screening will also ensure that the participant has ≥90°hip flexion range of movement bilaterally, knee flexion range ≥90° and full knee extension bilaterally, and that the hip quadrant test [28] is clear bilaterally. If groin pain on quadrant testing is greater than 5/10 on the Pain NRS, or the difference in pain levels between sides is greater than 2/10, the participant is excluded. Additionally, the participant must be able to flex the trunk forward with hands reaching at least to the knees with ≤2/10 back pain, and have adequate hip, knee and ankle mobility to be able to perform a squat to 60° flexion at the hips.

The participant will then be referred for MRI (if no contraindications e.g. cardiac pacemaker, metal implants etc.) and X-ray investigations at a participating radiology clinic, as a confirmed diagnosis of gluteal tendinopathy on MRI, based on a classification system from a previous study [11] will also be required for eligibility. Tendinopathy will be defined as an intratendinous increase in signal intensity on T2-weighted images (Table 2). Participants must have no contraindications to MRI (e.g. cardiac pacemaker, metal implants etc). An X-ray (AP and Lateral) is required to grade osteoarthritis severity using the Kellgren-Lawrence Scale [29]. Those with a score of >2 will be excluded from the study. To minimize unnecessary radiation exposure, if the patient has had previous appropriate X-rays within the last 6 months, they will not require a second lateral hip X-ray.

**Table 2:** MRI Image Analysis – Classification of Pathology for definition of gluteal tendinopathy

T2 Hyperintensity around Greater Trochanter (representing oedema/fluid)	
Size	(1) Tiny (thin slit of fluid)
	(2) Small (localized, mild distension)
	(3) Medium (localized, moderate distension)
	(4) Large (localized, marked distension)
Shape	(1) Feathery
	(2) Crescentic
	(3) Round (distended bursa)
Location	(1) Subtendinous
	(2) Intratendinous*
	(3) Subfascia lata
	(4) Superficial to fascia lata
Partial thickness tear	Tendon irregular, thinned or focally discontinuous
Full thickness tear	Discontinuity and/or retraction of the torn tendon from greater trochanter

*Intratendinous high T2 signal considered as tendinopathy with a thickened tendon without any irregularity, tendon thinning, or focal tendon discontinuity

#### Concealed allocation

A computerised randomisation schedule stratified for site (Brisbane, Melbourne) will be prepared by an independent off-site body (The Berghofer Queensland Institute of Medical Research, Clinical Trial and Biostatistics Unit). To conceal randomisation, consecutively numbered, sealed, opaque envelopes will be prepared by a research assistant not involved in recruitment, and kept in a locked cabinet accessible only to the assistant. These envelopes will be opened at each site sequentially once the participant has been confirmed into the study by MRI, and all baseline measurements have been completed. If allocated into the CSI group, an appointment will be made for the participant to receive an US-guided injection, administered by an experienced Radiologist/Sports Physician. If allocated into the exercise and load management group or into the group that will adopt the wait and see approach, an appointment will be made for the participant to attend a trial physiotherapy clinic.

#### Blinding

The investigator assessing the outcome measures will be blind to group allocation, and will not be involved in any of the interventions. Participants will be informed in writing and verbally that they have an equal chance of being allocated into one of the three groups (CSI, exercise and load management, wait and see). They will not be made aware of the study hypotheses. Participants will be requested not to divulge any details about their treatment group to the investigator involved in assessing the outcome measures, and will be reminded of this prior to each encounter. It is not possible to blind the patients, physiotherapists providing the treatment, the radiologist administering the CSI, nor the physiotherapists providing the basic information to the wait and see group. Statistical analysis will be conducted on a blinded intention-to-treat basis.

#### Interventions

*Corticosteroid injection:* Participants allocated to this group will attend the clinic of the experienced radiologist or sports physician who will be performing the injection. Seven specialist radiologists are participating in this trial, with a range of 15–40 years of experience. The injection will be performed at the same radiology clinic that the participant attended to have their MRI and XRay investigations performed. After standard preparation procedures, the participant will be positioned on the table with the affected side raised. A preliminary ultrasound scan will be performed with pre procedural images taken in a longitudinal plane. The depth of injection required will be measured and area of maximal tenderness marked. A linear probe which is appropriate for the participant body habitus (i.e. 12 MHz) will be cleaned using sterile wipes, and the appropriate syringe and needle selected. A further preliminary scan will be performed, and the radiologist washes their hands and gloves up. The steroid (Celestone 1 ml or Kenacort A40 1 ml (depending on availability)) and local anaesthetic (Bupivacaine 2 ml or Marcaine 1 ml) will be drawn up using sterile techniques and the participant’s skin and ultrasound probe will be cleansed with chlorhexidine and the area draped. The radiologist will place the needle through the superficial structures until it reaches the bursa following which the steroid and local anaesthetic mixture will be injected widely in different directions through the bursa. On completion of the procedure, a dressing will be applied to the puncture site. A post procedural check will be performed, and a further explanation of what to expect and basic advice on tendon care will be given to the participant. A weekly diary will be completed, recording levels of pain and function and any adverse effects.

*Exercise, education and load management programme:* Participants allocated to this group will be prescribed a home exercise programme to be performed daily, which will be limited to four to six exercises (to optimise adherence), and should take no longer than 15–20 min to complete. Participants will also receive more specific and detailed advice and education on tendon care. This information will be delivered in hard copy handouts, verbal explanation and DVD. They will attend the physiotherapy clinic for individual supervised exercise sessions, and be treated by an experienced and registered musculoskeletal physiotherapist. Treating practitioners will have attended a training session outlining the study objectives and requirements, demonstrating the exercise protocol and progressions, as well as the detailed education material, and will be expected to be proficient in the intervention. They will also be provided with a detailed study manual for reference. The physiotherapy sessions will be once a week for the first 2 weeks, then twice a week for the next 6 weeks. The first session will be 1 h long, in order to perform detailed education and demonstration of the exercises. Successive sessions will be 30 min in duration, and include a brief interview and physical assessment to gauge the response to the exercise and load management program, modify or progress the home exercises, and supervise a twice weekly heavier loading exercise programme.

**Fig. 1:**
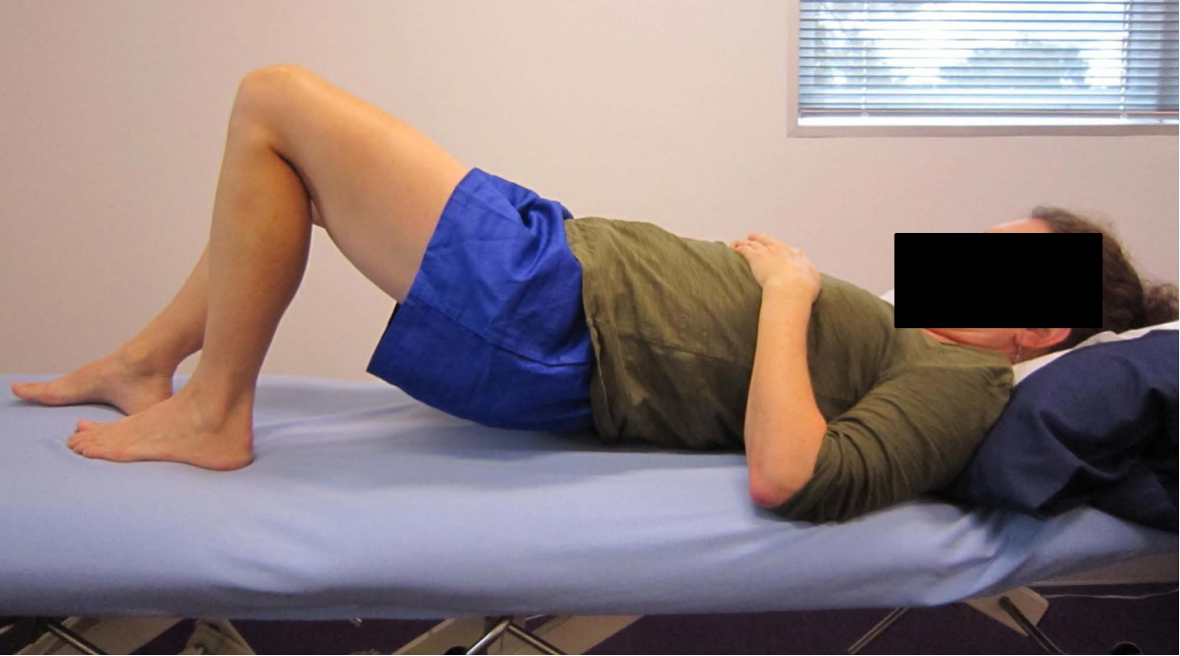
Offset bridging exercise

**Fig. 2:**
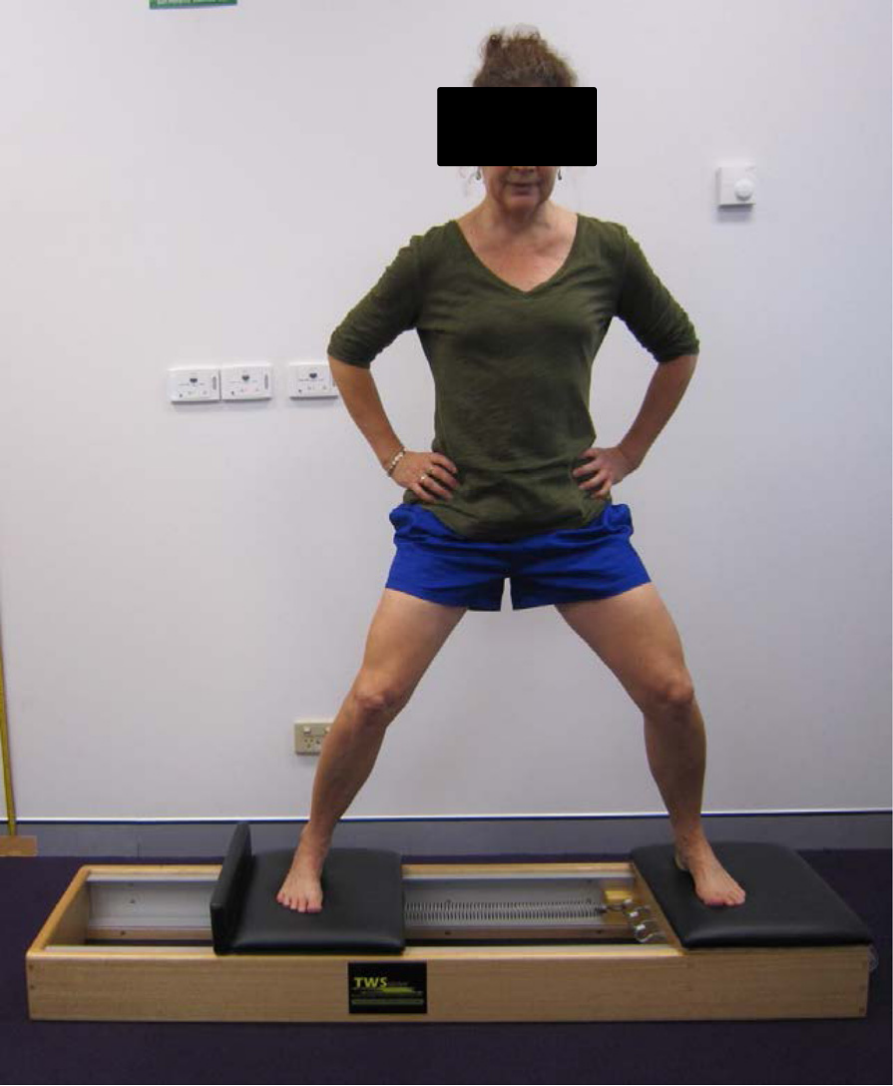
Spring resisted abduction on a sliding platform

The exercises will include functional retraining, and targeted strengthening for the hip and thigh muscles, with a particular focus on the hip abductor muscles, and dynamic control of adduction during function. Exercise difficulty will be gradually increased as tolerated by the participant, in order to optimise improvements in muscle strength and function without significant aggravation of the participant’s pain. Difficulty level will be monitored with the Borg Scale [30] where warm up will be performed at a light level (Borg 11–12), functional retraining at a somewhat hard to hard level (Borg 13–15), and the slow heavy targeted strengthening moving from somewhat hard towards the hard to very hard level (14–17), depending on the participants response to loading. No change in pain over the greater trochanter will be acceptable during functional retraining, as this may indicate inadequate alignment control, and excessive compressive tendon loading. As the heavy slow strengthening exercises avoid tendon compression completely, a maximum of NRS 5/10 pain will be tolerated as long as this eases afterwards and does not result in increased pain levels that night or the next morning. Participants’ responses to the exercises will be closely monitored, and loading levels adjusted as required to prevent any increases in pain from week to week. Table 3 outlines the clinic and home based exercise protocols, and Figs. 1 and 2 show some examples of the prescribed exercises.

**Table 3:** Exercise Dosage and Progressions

Stage	Exercise	Effort	Speed	Reps	Sets	Freq
Week 1- Familiarisation	*Low load activations*	Light	Slow onset	10	1–2	BD
	Static Abduction:		Hold 5–10 s			
	In supine lying	Light	Slow onset	3–5	1	BD
	In standing		Hold 5–15 s			
	*Pelvic Control during Functional Loading:*					daily
	Bridging	Light	Moderate	10	1	daily
	Double Leg Bridging					
	Functional Strengthening:	Light- SWH	Slow	10	1	
	Double leg squats					
	*Abductor Loading* via *Frontal Plane Movement:*	Light	Moderate	10 each	1	daily
	Sidestepping					
Week 2 – Early Loading & Movement Optimisation	*Low load activations*	Maintain as per week 1				
	Static Abduction:					
	*Pelvic Control during Functional Loading:*					
	Bridging:					
	Double leg bridging	Light	Slow	10	1	daily
	Single leg biased ex:	SWH	Slow	5	1	
	Offset bridging					
	Functional Strengthening:					
	Double leg squats	Light	Slow	10	1	daily
	Single leg biased ex:	SWH	Slow	5	1	
	Offset squat					
	*Abductor Loading* via *Frontal Plane Movement:*	Light	Moderate	15 each	1	daily
	Sidestepping					
Week 3–8 – Graduated Loading	*Low load activations*	Maintain as per week 1				
	Static Abduction:					
	*Pelvic Control during Functional Loading:*					
	Bridging:	Light	Slow	5	1	daily
	Double leg bridging					
	Single leg biased ex	SWH – Hard		5–10	2	daily
	Functional Strengthening:					
	Double leg squats	Light	Slow	5	1	
	Single leg biased ex	SWH - Hard		5–10	2	
	*Abductor Loading* via *Frontal Plane Movement:*					daily
	Sidestepping	Light	Moderate	10 each	1	
	Band Sideslides	SWH- Hard		5–10 each	1–2	
Week 3–8 – Graduated Loading; Sliding platform with spring resistance	All supervised by Physiotherapist in Clinic					
*Warm up*	*Abductor Loading* via *Frontal Plane Movement:*					
	Bilateral Abduction:					Twice weekly
	In upright	Light	Moderate	5 each way	1	
	In minisquat	Light	Moderate	5 each way	1	
*Higher level loading*	*Abductor Loading* via *Frontal Plane Movement:*					Twice weekly
	Bilateral Abduction:					
	In upright	SWH-VH	Slow	5–10 each way	1	
	In minisquat	SWH-VH	Slow	5–10 each way	1	
	*Pelvic Control during Functional Loading:*	Light - SWH	Moderate	5–10	1–2	Twice weekly
	Scooter					

Repetitions (Reps); Frequency (Freq); Effort based on Borg Scale (Borg, [30]); Somewhat Hard (SWH); Very Hard (VH); Speed: Slow = 3 s each movement phase – up/down/in/out; Moderate = 2 s each movement phase; Bi-daily (BD)

Adherence to the exercise programme will be monitored by the physiotherapists and with the participants also completing a daily diary, outlining the exercises completed (number of sets and repetitions), any adverse responses and action taken. If any adverse event occurs during the treatment period, the participants will also be encouraged to report directly to their physiotherapist (e.g. worsening of pain, new symptoms or any injury). Any reports of adverse events will be recorded in the treatment notes and diary. The diary will be collected from the participant at the 8 week follow up assessment.

*Wait and see:* Participants allocated to adopt a wait and see approach will attend one session with a trial physiotherapist, where they will receive reassurance that the condition is likely to resolve over time, as well as advice regarding general tendon care and self-management. They will also receive a standard information pamphlet about the condition and basic self-management. The therapists will take time to answer any questions about adopting a wait and see approach to ensure the participant is confident that this is an appropriate and sensible approach to adopt. Participants in this group will complete a weekly diary, outlining any problems that may have been encountered related to the study.

### Outcome measures

A battery of primary and secondary outcome measures will be recorded. They are summarized in Table 4.

**Table 4:** Primary and Secondary Outcome measures

Primary outcomes	Measurement	Baseline	4 weeks	8 weeks	12 weeks	26 weeks	52 weeks
Average Pain over the last week	11-point Pain Numeric Rating Scale (NRS), with terminal descriptors of 0 = ‘no pain’ and 10 = ‘worst pain possible’	√	√	√	√	√	√
Perceived overall change in condition of Hip	Global Rating of Change Scale		√	√	√	√	√
Secondary Outcomes	Measurement						
Global Impact of pain	Lateral Hip Pain Questionnaire	√	√	√	√	√	√
Function	Patient Specific Functional Scale	√	√	√	√	√	√
Quality of life	EuroQoL	√	√	√	√	√	√
Muscle strength	Static painfree abductor muscle strength	√		√			
Muscle Function	Active Lag Abductor Muscles	√		√			
Pain Catastrophising	Pain Catastophising Scale	√	√	√	√	√	√
Depression	PHQ-9	√	√	√	√	√	√
Pain and Function	VISA-G	√	√	√	√	√	√
Pain Self-Efficacy	Pain Self-Efficacy Questionnaire	√	√	√	√	√	√
Physical Activity Levels	Active Australia Survey	√	√	√	√	√	√
Economic Costs	OCC-Q	√				√	√

*EuroQoL* European quality of life questionnaire, *PHQ-9* patient health questionnaire-9; *OCC-Q* osteoarthritis costs and consequences questionnaires

#### Primary outcome measures

There are two primary outcome measures: (1) Global Rating of Change Score, and (2) Average Pain over the previous week.

**Fig. 3:**
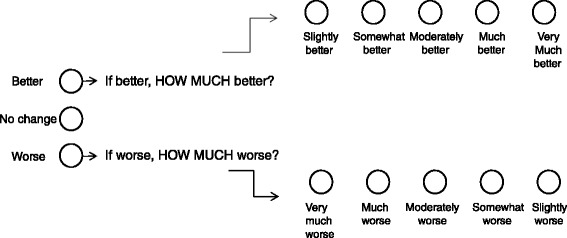
Primary Outcome Measure 1: Global Rating of Change Scale, modified from Kamper et al [31]

1. The Global Rating of Change Scale (GROC) is an 11-point scale in which the participant is asked to rate their perceived overall change in condition of their hip from the time that they began the study until the present, as Worse, No Change, or Better. If they indicate worse, the patient will then be asked *how much* worse on a five-point scale from Very Much Worse to Slightly Worse, and if they are better, they will be asked *how much* better on a five-point scale from Slightly Better to Very Much Better [31] (Fig. 3). Measuring patient perceived change using scales such as the GROC scale has previously been shown to be clinically relevant and a stable concept for interpreting meaningful improvements from an individual perspective [32].
2. The average pain over the previous week is measured on an 11-point Pain Numeric Rating Scale (NRS), with terminal descriptors of 0 = ‘no pain’ and 10 = ‘worst pain possible’. The minimum important difference (MID) for the NRS has been found to be -1.5 points for musculoskeletal disorders [33].

#### Secondary outcome measures

To ensure we have sufficient information regarding treatment responses there are a battery of secondary outcome measures: (1) Hip abductor muscle strength, (2) The VISA-G questionnaire, (3) the Patient Specific Functional Scale (PSFS), (4) the Pain Self-Efficacy Questionnaire, (5) The Pain Catastrophising Scale, (6) the Patient Health Questionnaire 9 (PHQ9),(7) the Active Australia survey, (8) EuroQoL (EQ-5D™) and (9) modified Osteoarthritis Costs and Consequences Questionnaire (OCC-Q).

**Fig. 4:**
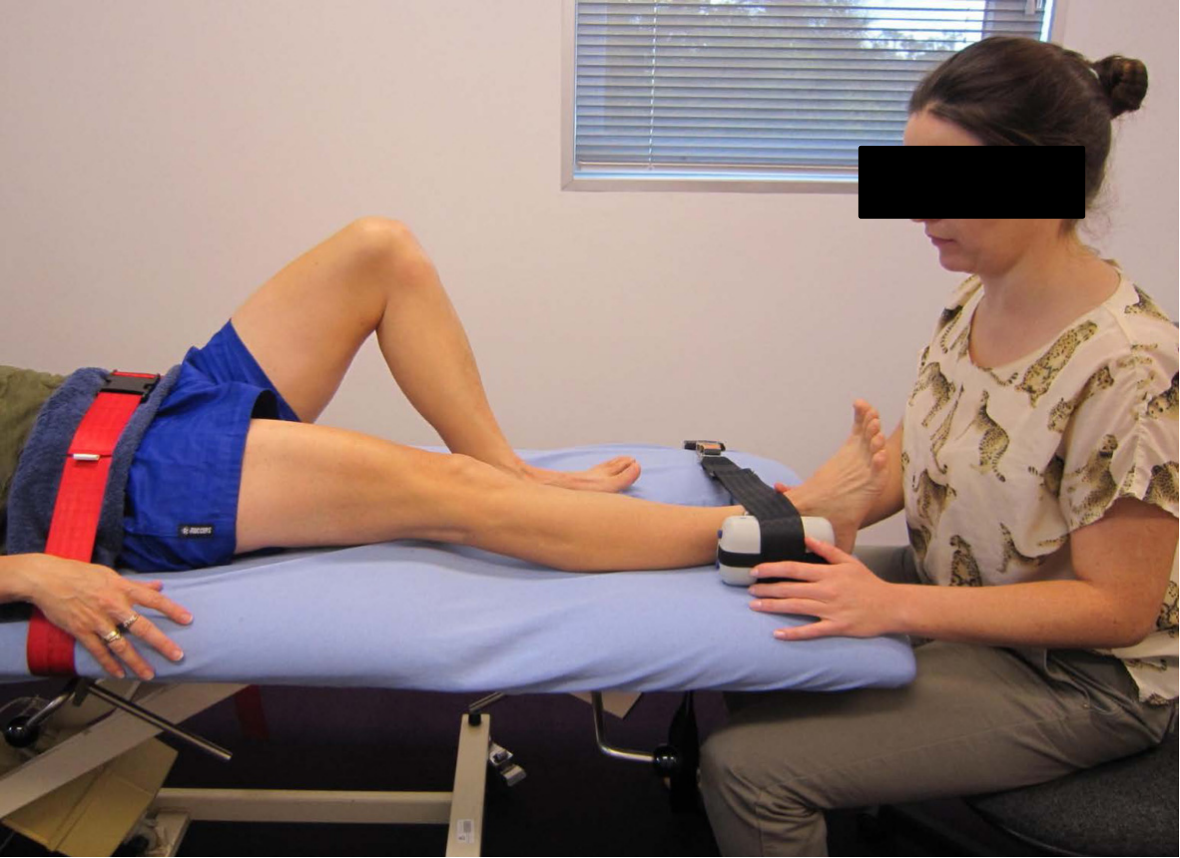
Measurement of maximum static abductor muscle strength

1. Hip abductor muscle strength will be measured with the participant in the supine position, with the tested leg extended and the hip at 10° abduction and 0° flexion, and the opposite leg flexed at the hip and knee. The pelvis will be stabilized with a seatbelt and towel, strapped to the plinth, and the participant will hold the side of the plinth with both hands. The centre of the dynamometer (Nicholas, Lafayett, IN47903 USA) will be positioned above the lateral malleolus of the fibula. Although such devices have been shown to have good to excellent reliability in different populations [34], a strap will be placed around the dynamometer and the plinth to stabilize the dynamometer and provide resistance to the abducting force, to eliminate the potential effect of examiner strength [35] (Fig. 4). The distance between the centre of the dynamometer and the most lateral aspect of the greater trochanter will be measured to calculate lever arm length. The supine position was adopted rather than side lying, as many people suffering from gluteal tendinopathy are unable to lie on the affected side due to pain. It has been found that the supine position also produces less measurement variation than the side lying position when testing hip abduction strength with a dynamometer [36]. Each participant will have one practice trial, followed by three experimental trials of hip abduction strength. To avoid pain aggravation, the participant will be asked to ramp up their force gradually, and then maintain a maximal contraction for 5 s. Peak force (Newtons) will be noted for each contraction, and the maximal value achieved over the three repetitions will be recorded. The distance between the greater trochanter and the mid-point of the dynamometer placement (proximal to the lateral malleolus) is then measured (m). Torque (Nm) is then calculated by the equation T = F (N) × D (m), and then standardized to body weight (Nm/kg). Standard verbal encouragement will be provided, with a consistent volume and level of enthusiasm. Fifteen seconds rest will be allowed between each contraction. A pain score will also be recorded for each repetition. This strength test has excellent test-retest reliability in our laboratory (ICC (95 % CI): 0.90 (0.83–0.95)).
2. The VISA-G questionnaire is a self-reported, patient-specific tool for evaluating the severity of disability in people with gluteal tendinopathy, and is modelled on other VISA questionnaires previously developed for Achilles and patellar tendinopathies, that have been shown to be valid and reliable tools for establishing severity of tendinopathy [37, 38]. This questionnaire consists of 8 items, addressing pain and function at the present time. Scores range from 0 to 100, with higher scores indicating less pain and better function. The VISA-G demonstrates good reliability and validity [39], providing an objective method for measuring changes in the severity of disability of people with gluteal tendinopathy.
3. The Patient Specific Functional Scale (PSFS) is a self-reported, patient-specific measure, designed to assess functional change in patients presenting with musculoskeletal disorders, and has been shown to be reliable, valid, and responsive to change in a number of musculoskeletal populations [40–42]. Patients are asked to identify three important activities they are unable to perform or are having difficulty with because of their problem. They are then asked to rate the current level of difficulty associated with each activity on an 11-point scale (where 0 is ‘unable to perform the activity’, and 10 is ‘able to perform at the same level as before the injury or problem’). Following intervention, the patients are then asked to rate the activities previously identified [43]. A total score is obtained by the sum of the activity scores, divided by the number of activities. Lower scores indicate greater functional difficulty. The MID has been found to be between 2.3 and 2.7 PSFS points in patients with musculoskeletal disorders of the lower extremity [33].
4. The Pain Self-Efficacy Questionnaire (PSEQ) [44] is a ten-item questionnaire used to assess the confidence that people with chronic pain have in performing activities while in pain. It covers a range of functions, including household chores, socialising, work, as well as coping with pain without medication. It takes 2 min to complete, has a high completion rate, can be used in assessment, treatment planning, and outcome evaluation [44], and has been shown to be a reliable and valid measure [45]. Participants are requested to rate how confidently they can presently perform the activities described on a seven-point Likert scale, where 0 = not at all confident and 6 = completely confident. The total score ranges from 0 to 60, where higher scores reflect stronger self-efficacy beliefs.
5. The Pain Catastrophising Scale is a 13-item self-report scale to measure pain catastrophising, and has been shown to be valid and reliable [46]. Participants are asked to reflect on past painful experiences and to indicate the degree to which they experienced each of 13 thoughts or feelings when experiencing pain, on 5-point scales with the end points (0) not at all and (4) all the time. The Pain Catastrophising Scale produces a total score, and three subscale scores assessing rumination, magnification and helplessness. The total score ranges from 0 to 52, with higher scores indicating higher levels of pain catastrophization. Pain catastrophising is said to relate to various levels of pain intensity reporting, physical disability and psychological disability in clinical and nonclinical populations.
6. The Patient Health Questionnaire 9 (PHQ-9) is a brief self-report tool for screening, diagnosing, monitoring and measuring the severity of depression. The nine item questionnaire determines the frequency of depressive symptoms over the past 2 weeks, where PHQ-9 scores of 5, 10, 15 and 20 represent mild, moderate, moderately severe and severe depression. In addition to making criteria-based diagnoses of depressive disorders, the PHQ-9 has been shown to be a valid and reliable measure of depression severity, and its brevity makes it a useful clinical and research tool [47]
7. The Active Australia Survey measures participation in leisure-time physical activity. The core questions apply to 1 week preceding completion of the survey, and consist of a short and reliable set of eight questions that can be easily implemented via telephone interviewing techniques or in face-to-face interviews. The Active Australia Survey has good reliability and validity and has been used in national surveys [48]. A number of different measures of participation in physical activity during the previous week can be obtained, including how many sessions of physical activity, total time and average time spent in each activity and ultimately the proportion of people who were doing sufficient activity to gain health benefits, or those who were sedentary.
8. The EuroQoL (EQ-5D™) is a standardised instrument for use as a measure of health-related quality of life. It is applicable to a wide range of health conditions and treatments, and provides a simple descriptive profile, where the participant indicates in tick boxes which statements about mobility, personal care, usual activities, pain and anxiety/depression best describe their health status, as well as a single index value for health status. The participant is asked to grade their current level of function in each dimension into one of 3° of disability (severe, moderate or none), and each health state is ranked and transformed into a single score, called the utility. This utility score is an expression of the Quality Adjusted Life Years (QALY), and is commonly used to make evidence-based decisions in analyses of cost-effectiveness [49]. It is designed for self-completion by respondents and is cognitively simple, taking only a few minutes to complete.
9. Economic costs data will be obtained from a modified version of the Osteoarthritis Costs and Consequences Questionnaire (OCC-Q). It is a self-administered questionnaire, which gives a broad representation of health care costs, and has been shown to be a feasible and valid method of capturing health care use and costs for patients with hip or knee pain compared with accessing administrative databases [50].

### Other measures

Several other measures will be included to provide additional information about participants, such as demographic information collected at phone interview and the initial physical screen. Participants in the CSI and wait and see groups will keep a weekly diary throughout the initial 8 weeks to record any adverse events and any co-interventions, including pain related medication use. This same information is captured via the daily diary in the physiotherapy group.

In addition to these measures the opportunity presents to test two novel condition-specific measures: (1) the Lateral Hip Pain Questionnaire (LHPQ) and (2) the hip abduction lag.

1. The LHPQ has been designed as a self-reported measure of pain and function with focus on issues specific to lateral hip pain sufferers. It has two primary subscales – one for Activities of Daily Living (ADL), and one for Sport. The ADL subscale encompasses questions related to pain (frequency, overall intensity, intensity for specific tasks, time to pain onset, and pain duration after sustained sitting), impact on function (overall and specific activities), and pain beliefs (fear of physical activity, and permanent impairment). The participant is asked to consider these aspects over the past week, and the total score ranges from 0 to 100, with higher scores indicating less pain and better function. The Sports subscale requires self-rating of pain (pain intensity while participating in sport, pain behaviour, time to pain onset) and impact on sporting participation. It is not completed if the participant does not compete in sport. Again, the time frame is considered over the past week, and the total score ranges from 0 to 100. The LHPQ is in a development phase, and will be tested alongside this main trial, and validated against information collected from other concurrent outcome measures (VISA-G) (Additional file 1).
2. Active lag of hip abduction (Active -Passive discrepancy) will be measured with the participant in the side-lying position, with the lower leg against the table in approximately 45° of hip flexion and 90° of knee flexion. The upper leg will be supported on pillows in a neutral position in the sagittal plane, with 10° abduction in the frontal plane to avoid compressive loading of the ITB over the greater trochanter. Rotation of the pelvis will be avoided, and a rolled towel will be placed under the waist angle to achieve a neutral lumbo-pelvic position. The assessor will stand behind the patient to ensure the pelvis is maintained in the starting position, and also stabilise the pelvis with a hand over the lateral iliac crest. A plurimeter (Australasian Medical & Therapeutic Instruments) will be placed on the distal femur, 5 cm proximal to the lateral joint line. The participant will be requested to actively abduct the hip to the maximal position that they are comfortably capable of, and this position will be recorded. The assessor will then passively abduct the hip to its end of range, stabilising the pelvis and supporting the lower leg, and this position will be recorded. A trial of this active and passive abduction movement will be performed first in order to ensure correct technique (hip in neutral flexion/extension and rotation), then this will be repeated three times. The difference between passive and active range of hip abduction is recorded as the Active Lag for each repetition, and the average of the three trials will be recorded for analysis.

### Procedure

**Fig. 5:**
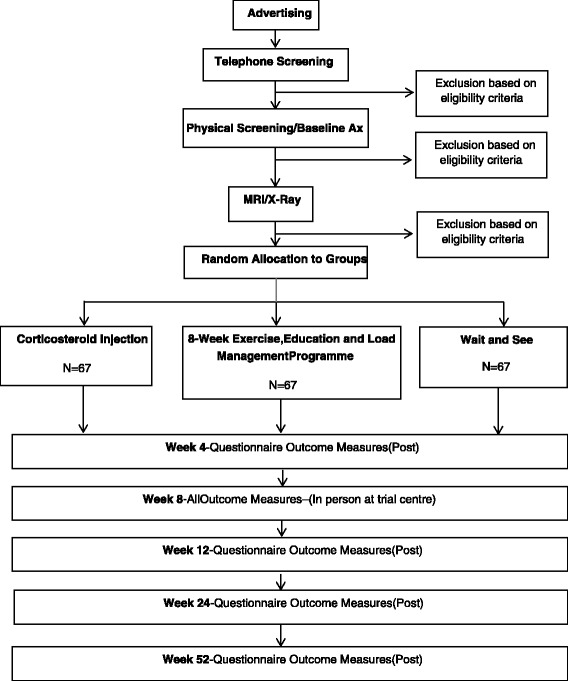
Flow of participants through RCT

The flow of participants through the study is outlined in Fig. 5. Following baseline testing and imaging, eligible participants will be randomly allocated into one of three groups: (1) CSI, (2) exercise, education and load management programme, or (3) wait and see. Participants will complete the questionnaire outcome measures at baseline and 4, 8, 12, 26 and 52 weeks after commencement of the study, and physical outcome measures (abductor muscle strength and lag) will be reassessed at 8 weeks. Regular contact will be maintained with participants via phone calls and emails to ensure completion and return of questionnaires at all time points. Participants will be asked to refrain from seeking other treatments during the study period, but analgesia and anti-inflammatory drugs will be permitted. All medication use and co-interventions will be recorded.

### Not per protocol treatments

All participants will be informed of the importance of following allocated treatments, but encouraged to record in their diary any deviations from protocol. Not per protocol treatments are for example, medications, other injections, other physiotherapy or treatment not specified by the allocation.

### Adverse events

Participants, physiotherapists and medical practitioners performing the treatments will report adverse effects to the research assistant who with the chief investigator will then ensure that if required the appropriate treatment for that adverse effect is undertaken and that the event is reported to the ethics committee. The participant will be monitored over the course of resolution.

### Data management

The responses to the GROC scale will be dichotomized, where “Success” will be defined as “Moderately better” to “Very Much Better”. The proportion of improved participants from each group will determine success of the intervention.

### Sample size

The treatment effect will be evaluated by comparing success rates on the primary outcome measure of the GROC score between groups. For the global rating of change score, sample size is based on the ability to detect a clinically relevant difference of 30 % in success rate between the two treatment groups and the control at eight weeks from baseline using the Dunnett’s test procedure. This sample size accounted for a 15 % loss to follow-up, a type I error rate of 0.05, any-pair power of 0.95 and all-pair power of 0.80. Assuming a success rate of 40 % for the control (the wait and see group), 70 % for the physiotherapy group and 70 % for the CSI group, the target sample size was calculated at 67 patients per group for a total sample size of 201 which is based on 2000 Monte Carlo sampling with the equivalence margin of 20 %. This sample size was chosen because a sample size of 61 per group was required for a point estimate of effect of two points on the 11 point pain numerical rating scale (the other primary outcome). The Clinical Trial and Biostatics Unit of the Berghofer Queensland Institute of Medical Research was responsible for calculating sample size.

### Statistical analysis

Statistical analyses will be conducted on a blinded intention-to-treat basis, with all participants who were initially randomised into the study included where data are available for each measurement time point.

The outcomes measured at baseline, 4, 8, 12, 26 and 52 weeks will be analysed using linear mixed and logistic regression models that will include their respective outcome measure scores as a covariate, participants as a random effect and treatment conditions as fixed factors. Variables such as age, sex, body mass index (BMI) and site will be included as covariates in the analysis. Regression diagnostics will be used to check for normality of the measures and homogeneity of variance where appropriate. Treatment mean contrasts will be defined a-priori: CSI versus load modification and exercise intervention, CSI versus wait and see approach, and load modification and exercise intervention versus wait and see approach. Alpha will be set at 0.05. Numbers needed to treat index will also be calculated.

The cost and utility data will be analysed in a manner consistent with the clinical outcome data. The resource use data captured by the OCC-Q will be valued using unit costs derived from local and national sources. If the cost data violate the assumptions of parametric statistics, non-parametric methods of analysing group means will be used. Non-parametric bootstrapping will be used for calculating means and confidence intervals. Estimates of the costs and effects of each treatment group in relation to the comparator and to each other will be presented, with sampling uncertainty. The incremental differences between these groups will be reported as incremental cost-effectiveness ratios (ICER), reported from both the societal perspective (primary) and the health system perspective (secondary), and will be presented in cost-utility scatter planes. Confidence intervals will be calculated for the ICERs. Sensitivity analyses will also be conducted to test the robustness of the models. Cost-effectiveness acceptability curves (CEACs) will also be calculated to determine the likelihood that the treatments can be considered cost-effective at the commonly accepted, policy-relevant willingness-to-pay (WTP) thresholds of one, two, and three times GDP per capita.

## Discussion

Despite its prevalence in the community and the associated disability that ensues, optimal management of gluteal tendinopathy has not been established. We propose that to effectively address the deficit in evidence for optimal management of gluteal tendinopathy, an RCT that compares commonly prescribed treatments, such as CSI, adopting a wait and see approach, and physiotherapy is required. In addition to this, current evidence suggests that management of tendinopathy needs to be targeted to the tendon and in that regard: (a) the diagnosis should involve a combination of clinical examination and MRI confirmation of tendon involvement, (b) an injection should be guided by imaging so as to be specific [51] and (c) any exercise be undertaken as part of a load management approach, which is now being recommended as the frontline treatment for managing tendinopathy [15].

Identification of the appropriate patient population in an RCT is critical both for the targeted applications of treatment and applicability of the study results to patients in the clinic. A patient presenting with lateral hip pain might have a number of musculoskeletal conditions acting as the potential source of pain around the lateral hip area. In addition to hip joint pathology, such as osteoarthritis, inflammatory arthritis, avascular necrosis or infection, other extra-articular causes of lateral hip pain reported in the literature include femoral neck stress fractures, spinal referred pain, nerve entrapment and tumours [3]. A strength of our proposed RCT is that we will use a combination of both clinical tests and MRI diagnosis that implicate gluteal tendinopathy as our key selection criteria. This is crucial to the outcomes of the trial, as the injection and the exercise and load management programme are specifically designed to manage and treat the condition of gluteal tendinopathy, rather than other hip conditions.

Corticosteroid injections are widely used in the management of many tendinopathies [20] including gluteal tendinopathy. One of the reasons they are widely used is likely due to the remarkable improvement in pain reported by patients in the first 4–8 weeks. The review by Coombes et al identified that only for lateral epicondylalgia of the elbow has there been evidence from several high quality RCTs that show that this early good effect is followed by delayed recovery when compared to adoption of a wait and see approach. Also, substantially higher recurrence rates were seen with CSI compared to when a wait and see approach or manual therapy and exercise was adopted. Notwithstanding these poor long-term outcomes, the likelihood of serious adverse effects of a CSI were rare and relatively minor in nature [20], and usually only include increased or radiating pain, local skin irritation or swelling, local soft tissue atrophy, infection or depigmentation [52]. It is likely that the combination of few adverse effects and a high proportion of favourable responses in the short term is one of the reasons for the continued use of these injections. We will minimise adverse events in our study through the use of well-trained medical practitioners who will utilise image guidance, as well as providing post-injection advice to manage load and its re-introduction in a graduated manner over the ensuing 6–8 weeks. That is, the patient will be warned that they could experience a recurrence if they overload the tendon too quickly after the injection and that there would be a temptation to do so as their pain would be much better during this period.

Participants allocated into the wait and see approach group will attend a single session with a trial physiotherapist, who will describe the condition and its development, provide reassurance that the condition will spontaneously improve over time, and advise a sensible approach to continued safe, non-painful activity. A number of RCTs comparing treatment approaches for lateral elbow tendinopathy have shown that by 12 months there is good resolution of the condition and no significant differences in outcome measures (e.g. pain, patient satisfaction, global improvement) between a wait and see group, CSI and physiotherapy [21, 53]. An RCT that compared CSI and usual care (analgesics as needed) in a population of people with greater trochanteric pain syndrome also found no significant differences in recovery (61 and 60 % totally or strongly recovered) and pain at rest or with activity between the two groups at 12 months [19]. Thus, despite a slower resolution, in the long term the wait and see approach is often superior to a CSI as outcomes are better and there is a lower recurrence rate (as observed in lateral elbow tendinopathy) [21].

Exercise and load management is proposed to be the cornerstone of an effective non-operative approach to tendinopathy [15]. We have chosen to investigate an exercise and load management approach that focuses specifically on hip abductor muscle function and avoidance of compressive loads on the gluteal tendons. The exercise programme avoids commonly adopted/prescribed hip muscle stretching techniques, because they are likely to place high compressive load on the tendons. It also limits hip adduction in the exercises that are used to facilitate and condition the gluteal muscles. The gluteal exercises are commenced at low loads that are gradually progressed over the course of treatment. The education element of this program reinforces the attention required to avoid or minimise compressive loading of the tendons. It does this by showing the patient through multimedia and personal instruction ways in which to minimize compressive loading of the tendons during everyday activities. We propose that this will provide the best circumstances under which to test conservative management against CSI and the adoption of a wait and see approach, both in the short and long term for both recovery and recurrence rates.

In order to optimise the rigor of the RCT and to minimise bias, a number of methodological factors have been incorporated into the design of the study. The study participants will be randomly allocated into the intervention groups via concealed allocation, as inadequately concealed allocation has been associated with bias in RCTs [54]. Due to the nature of the interventions, it is not possible to blind the participants or the treating therapists to the allocated groups.

In further attempts to reduce bias, data will be analysed on an intention-to-treat basis, which preserves the randomisation process and also imitates the real life situation where the possibility exists that not all participants receive their prescribed treatment. Additionally, the statistical analysis will be conducted blind to treatment group allocation.

The RCT will utilise outcome measures that have established reliability and validity, as recommended by the CONSORT group [55]. In addition to improving measurement quality and outcomes, it also enables direct comparisons with other studies that investigate conservative management of gluteal tendinopathy and possible meta-analyses. The outcome measures are easily administered in a clinical setting, which will enhance the relevance and application of study findings to clinicians.

## Conclusion

An evidence-based, effective, appropriate conservative management strategy for gluteal tendinopathy that provides both short- and long-term benefits in terms of reduction of pain and improvement in function is needed. This RCT will implement high-quality methodologies in accordance with CONSORT guidelines. It is anticipated that findings from this study will contribute to the body of evidence-based practice available to clinicians in order to provide effective management of gluteal tendinopathy.

## Ethics and consent to participate

The trial adheres to the principles of the Declaration of Helsinki with ethical approval obtained from the Human Research Ethics Committee of the University of Queensland (HREC No. 2012000930) and the Behavioural and Social Sciences Human Ethics Sub-Committee of the University of Melbourne (Ethics ID 1238598). All participants will provide written informed consent.

## Consent to publish

Not applicable.

## Availability

This is a study protocol, and this trial is presently in progress. Thus, no data are currently available.

## Additional file

Additional file 1: Lateral Hip Pain Questionnaire. (PDF 336 kb)

## Abbreviations

ADD: passive hip adduction in side lying
ADL: activities of daily living
AP: anteroposterior
ASIS: anterior superior iliac spine
Ax: assessment
BD: bidaily
BMI: body mass index
CEAC: cost effectiveness acceptability curve
CI: confidence interval
CSI: corticosteroid injection
EQ-5D™–EuroQol: European quality of life questionnaire
FABER: flexion, abduction, extenal rotation test for the hip
FADER: flexion, adduction, external rotation test for the hip
Freq: frequency
GDP: gross domestic product
GMed: gluteus medius
GMin: gluteus minimus
GROC: global rating of change
H1: hypothesis 1
H2: hypothesis 2
ICC: intraclass coefficient
ICER: incremental cost effectiveness ratio
IR: internal rotation
ITB: iliotibial band
LHPQ: lateral hip pain questionnaire
MID: minimum important difference
MRI: magnetic resonance imaging
NHMRC: National Health and Medical Research Council
NRS: numeric rating scale
OCC-Q: osteoarthritis costs and consequences questionnaire
PCS: pain catastrophizing scale
PHQ-9: patient health questionnaire 9
PSEQ: pain self efficacy questionnaire
PSFS: patient specific functional scale
QALY: quality adjusted life years
RCT: randomised clinical trial
Reps: repetitions
SLS: single leg stance
SWH: somewhat hard
SWT: shock wave therapy
TAC: transport accident commission
US: ultrasound
VH: very hard
WTP: willingness to pay
