# Supplementary material for: Exercise and load modification versus corticosteroid injection versus ‘wait and see’ for persistent gluteus medius/minimus tendinopathy (the LEAP trial): a protocol for a randomised clinical trial
Source: BMC Musculoskelet Disord. 2016 Apr 30;17:196. doi: 10.1186/s12891-016-1043-6 (PMC4852446; doi:10.1186/s12891-016-1043-6)
Supplement: Additional file 1: — Lateral Hip Pain Questionnaire. (PDF 336 kb) [file 12891_2016_1043_MOESM1_ESM.pdf]

Name/ID: ________________________________ **Date: __________**

THE UNIVERSITY OF QUEENSLAND

Lateral Hip Pain Questionnaire

The lateral hip pain questionnaire will help us understand how your hip pain is impacting on your life. Please note you will be answering ALL questions in Part A related to everyday tasks. If you are currently not doing any of these tasks, please estimate how your hip pain would respond. You will only need to answer Part B if you usually participate in sport.

PART A: ACTIVITIES OF DAILY LIVING (Please circle your answer to each question)

Pain Subsection:

1. Pain Intensity: Overall. Please rate the average amount of hip pain you have had **over the past week**, where **zero** (0) means that you had **no pain**, and **ten** (10) means you had the **worst pain imaginable**.

**No Pain**										**Worst Imaginable**
0	1	2	3	4	5	6	7	8	9	10

2. Pain Intensity: Specific tasks. This question relates to the intensity of the pain you experience during a number of specific activities. You must provide an answer for each line. If you are not currently doing any of these tasks, please *estimate* what your pain would be. Indicate the **average level of hip pain** you have had **over the past week** by circling the number that best describes your pain on a scale of zero to ten, as above.

**Activity**	**No Pain**									**Worst Imaginable**
**Lying on your most painful side**	0	1	2	3	4	5	6	7	8	9	10
**Rising from sitting**	0	1	2	3	4	5	6	7	8	9	10
**Standing on 1 leg to dress**	0	1	2	3	4	5	6	7	8	9	10
**Walking at a normal pace**	0	1	2	3	4	5	6	7	8	9	10
**Walking at a fast pace**	0	1	2	3	4	5	6	7	8	9	10
**Climbing stairs**	0	1	2	3	4	5	6	7	8	9	10

3. Pain Frequency: Please estimate how often your pain has been present **over the past week**, by circling the percent of time that best describes your current situation. **Zero percent (0%)** means you **did not have any pain** at all, and **one hundred percent (100%)** means you have had **constant pain**.

	**Not at all**										**Constant**
	0%	10%	20%	30%	40%	50%	60%	70%	80%	90%	100%
Office Use: Score:	0	1	2	3	4	5	6	7	8	9	10

4. Pain Onset: Please indicate the **average length of time** it took **before you started feeling pain** (or an *increase* in pain) for each of these activities, **over the past week**. Circle the length of time for each activity.

							Score:
**Lying on your most painful side**	No Pain	More than 60 minutes	31 to 60 minutes	11 to 30 minutes	1 to 10 minutes	Less than one minute	
**Walking at normal pace**	No Pain	More than 60 minutes	31 to 60 minutes	11 to 30 minutes	1 to 10 minutes	Less than one minute	
**Walking at fast pace**	No Pain	More than 60 minutes	31 to 60 minutes	11 to 30 minutes	1 to 10 minutes	Less than one minute	
Office Use: Score:	0	2	4	6	8	10	

5. Duration of pain & stiffness after rising from sitting: **After** you have been **sitting for longer than 30 minutes**, when you rise and walk off, **how long do you have hip pain or stiffness for** (or *increased* hip pain if your pain is constant)? Please indicate the length of time by circling a box below.

	No Pain	Less than 10 seconds	10 seconds to 2 minutes	2 to 5 minutes	5 to 10 minutes	Longer than 10 minutes
Office Use: Score:	0	2	4	6	8	10

Function Subsection:

6. Rate how much impact your hip has had overall on your normal daily activities over the past week, where **zero (0) is no impact, and ten (10) is maximum impact** so that you are unable to perform any normal activities such as work, household activities (cooking,cleaning), personal activities (dressing, washing), and general walking.

**No impact**										**Maximum Impact**
0	1	2	3	4	5	6	7	8	9	10

7. Please indicate the **degree of difficulty (NOT PAIN)** you experienced due to your hip pain for each of the following activities over the past week, where **zero** (0) means you are having **no difficulty**, and **ten** (10) means you are **unable to perform the task at all.**

**Activity**	**No Difficulty**										**Unable To Do**
**Rising from sitting after 30 minutes**	0	1	2	3	4	5	6	7	8	9	10
**Climbing one flight of stairs**	0	1	2	3	4	5	6	7	8	9	10
**Lifting painful leg out of the car**	0	1	2	3	4	5	6	7	8	9	10
**Standing on one leg eg to dress**	0	1	2	3	4	5	6	7	8	9	10

8. For how long can you walk at normal pace before you are stopped by your hip pain: (Please circle)

	Not limited	More than 60 minutes	31 to 60 minutes	11 to 30 minutes	1 to 10 minutes	Less than one minute
Office Use: Score:	0	2	4	6	8	10

Impact Subsection:

9. Please **rate your agreement** with the following statement:
I'm afraid that I might injure myself if I do any physical activity

**Strongly Disagree**										**Strongly Agree**
0	1	2	3	4	5	6	7	8	9	10

10. In your view, how large is the risk that your pain may become permanent where **zero (0) is no chance and ten (10) is** what you believe to be **a definite outcome**?

**No chance**										**Definitely**
0	1	2	3	4	5	6	7	8	9	10

Office Use:

LHPQ ADL Score : Pain subscale (/120) + Function subscale (/60) + Impact subscale (/20) = **/100**

PART B: SPORTS PARTICIPATION (Please circle your answer to each question)

Only answer questions in this section if you participate in sport. If you usually participate in a number of sports, answer the question with respect to the sport in which your hip pain causes most problems. If you are not currently participating in your normal sport because of your hip pain, please estimate how your hip pain would respond.

11. Please rate the **average amount of pain** you have been experiencing in your hip **over the past week**, while participating in sport, or estimate how your hip would respond. **Zero** (0) means that you had **no pain**, and **ten** (10) means you had the **worst pain imaginable**.

**No Pain**										**Worst Imaginable**
0	1	2	3	4	5	6	7	8	9	10

12. Please indicate the **average length of time it took your pain to come on** while participating in sport, **over the past week**. If you did not participate in your normal sport in the last week, please estimate how your hip pain would respond.

	No pain	Longer than 60 minutes	31 to 60 minutes	11 to 30 minutes	1 to 10 minutes	Less than one minute
Office Use Score:	0	2	4	6	8	10

13. **Over the past week**, how long does your hip pain last (or aggravation of your hip pain if your pain is constant) after sport? Rate the sport that is most problematic for your hip pain.

	No Pain	Less than 10 minutes	11 minutes to 2 hours	More than 2 hours, but settled by the next morning	Pain is present the next morning	Pain lasts more than 24 hours
Office Use: Score:	0	2	4	6	8	10

14. Please rate the **degree of difficulty (NOT PAIN)** you experienced due to your hip pain while participating in sport over the past week, where **zero** (0) means you are **having no difficulty**, and **ten** (10) means you are **unable to participate at all**.

**Activity: Write in your sport below**	**No Difficulty**										**Unable To Do**
	0	1	2	3	4	5	6	7	8	9	10

15. Please rate your agreement with the following statement:

This injury may permanently prevent me from continuing or returning to my sport.

**Strongly Disagree**										**Strongly Agree**
0	1	2	3	4	5	6	7	8	9	10

Office Use:

LHPQ Sport Score: (/50) x2 = **/100**
